# Predictors and Variability of Repeat Measurements of Urinary Phenols and Parabens in a Cohort of Shanghai Women and Men

**DOI:** 10.1289/ehp.1306830

**Published:** 2014-03-21

**Authors:** Lawrence S. Engel, Jessie P. Buckley, Gong Yang, Linda M. Liao, Jaya Satagopan, Antonia M. Calafat, Charles E. Matthews, Qiuyin Cai, Bu-Tian Ji, Hui Cai, Stephanie M. Engel, Mary S. Wolff, Nathaniel Rothman, Wei Zheng, Yong-Bing Xiang, Xiao-Ou Shu, Yu-Tang Gao, Wong-Ho Chow

**Affiliations:** 1 Department of Epidemiology, UNC Gillings School of Global Public Health, Chapel Hill, North Carolina, USA; 2 Department of Medicine, Division of Epidemiology, Vanderbilt University School of Medicine, Nashville, Tennessee, USA; 3 Division of Cancer Epidemiology and Genetics, National Cancer Institute, National Institutes of Health, U.S. Department of Health and Human Services, Bethesda, Maryland, USA; 4 Department of Epidemiology and Biostatistics, Memorial Sloan-Kettering Cancer Center, New York, New York, USA; 5 Division of Laboratory Sciences, National Center for Environmental Health, Centers for Disease Control and Prevention, Atlanta, Georgia, USA; 6 Department of Community and Preventive Medicine, Mount Sinai School of Medicine, New York, New York, USA; 7 Shanghai Cancer Registry, Shanghai Cancer Institute, Shanghai, China

## Abstract

Background: Exposure to certain phenols is ubiquitous because of their use in many consumer and personal care products. However, predictors of exposure have not been well characterized in most populations.

Objectives: We sought to identify predictors of exposure and to assess the reproducibility of phenol concentrations across serial spot urine samples among Chinese adults.

Methods: We measured 2,4-dichlorophenol, 2,5-dichlorophenol, butyl paraben, methyl paraben, propyl paraben, benzophenone-3, bisphenol A, and triclosan in urine collected during 1997–2006 from 50 participants of the Shanghai Women’s Health Study cohort and during 2002–2006 from 50 participants of the Shanghai Men’s Health Study cohort. We investigated predictors of concentrations using the Satterthwaite *t*-test, and assessed reproducibility among serial samples using intraclass correlation coefficients (ICCs) and Spearman correlation coefficients (SCCs).

Results: Creatinine-corrected phenol concentrations were generally higher among women than men. Participants who had taken medicine within the previous 24 hr had higher concentrations of propyl paraben. Cigarette smoking was associated with lower concentrations of propyl and methyl parabens among men. Bottled water consumption was associated with higher bisphenol A, 2,4-dichlorophenol, and 2,5-dichlorophenol concentrations among women. Among men, reproducibility across serial samples was moderate for 2,4-dichlorophenol and 2,5-dichlorophenol (ICC = 0.54–0.60, SCC = 0.43–0.56), but lower for other analytes (ICC = 0.20–0.29). Reproducibility among women was low (ICC = 0.13–0.39), but increased when restricted to morning-only urine samples.

Conclusions: Among these 100 Shanghai residents, urinary phenol concentrations varied by sex, smoking, and consumption of bottled water. Our results suggest that a single urine sample may be adequate for ranking exposure to the precursors of 2,4-dichlorophenol and 2,5-dichlorophenol among men and, under certain circumstances, among women.

Citation: Engel LS, Buckley JP, Yang G, Liao LM, Satagopan J, Calafat AM, Matthews CE, Cai Q, Ji BT, Cai H, Engel SM, Wolff MS, Rothman N, Zheng W, Xiang YB, Shu XO, Gao YT, Chow WH. 2014. Predictors and variability of repeat measurements of urinary phenols and parabens in a cohort of Shanghai women and men. Environ Health Perspect 122:733–740; http://dx.doi.org/10.1289/ehp.1306830

## Introduction

Although human exposure to phenols (or their precursors) and parabens is ubiquitous in western industrialized countries, little is known about the patterns and predictors of exposure to these compounds in Asia or about their changes over time. One phenol that has received particular attention is bisphenol A (BPA), used to manufacture resins and polymers found in container linings and dental sealants. Other commonly used phenols include benzophenone-3, used in sunscreen; triclosan, a microbicide used in many household and personal care products; and parabens, used as preservatives in many personal care products. Of interest is 2,5-dichlorophenol, a metabolite of 1,4-dichlorobenzene, which is used in mothballs and room deodorizers.

These phenols are of interest because of hormonal and other biological effects observed *in vitro* and *in vivo.* Effects include weak-to-moderate estrogenicity and antiandrogenicity *in vitro* (Fang et al. 2000), which may disrupt the endocrine system in humans, and adverse reproductive outcomes, including low birth weight and altered reproductive endocrine parameters (Savabieasfahani et al. 2006), carcinogenicity (National Toxicology Program 2011), and hematological abnormalities (Aiso et al. 2005) in animal models. Determining whether these chemicals cause these or other adverse health effects in humans at typical exposure levels is important.

These phenols pose a challenge for exposure assessment in epidemiologic studies because they are rapidly eliminated from the body, with half-lives of hours [Centers for Disease Control and Prevention (CDC) 2009]. This is of particular concern for prospective epidemiologic studies of diseases with long latency, such as cancer, where estimates of exposure dating back years to decades may be important and where often only a single prediagnostic urine sample is available. Any temporal variability in exposure would be influenced by behavioral changes (in the use of, or exposure to, products) and by secular changes in the composition or selection of products available on the market, which could affect the reliable estimation of individuals’ absolute or relative exposures.

Several studies that examined variability in urinary concentrations over weeks to months reported generally low reproducibility for BPA (Braun et al. 2011, 2012; Mahalingaiah et al. 2008; Nepomnaschy et al. 2009; Teitelbaum et al. 2008) and fair reproducibility for other phenols and parabens (Meeker et al. 2011; Smith et al. 2012; Teitelbaum et al. 2008) among U.S. children and adults. However, the temporal variability of urinary concentrations of these chemicals either among Asians or over years has not previously been described. The aims of the present study were to identify predictors of exposure to selected phenols and to determine the reproducibility of phenol concentrations across serial urine samples collected over several years among urban Chinese adults.

## Methods

*Study population*. The Shanghai Women’s Health Study (SWHS) and Shanghai Men’s Health Study (SMHS) are population-based, prospective/cohorts of 75,000 women (40–70 years of age at baseline) and > 61,000 men (40–75 years of age at baseline) living in urban Shanghai (Cai et al. 2007; Zheng et al. 2005). Recruitment occurred during 1997–2000 for the SWHS and 2002–2006 for the SMHS. In both studies, eligible participants were identified from a roster of all community residents and all provided informed consent; participation rates were 92% in the SWHS and 74% in the SMHS. A physical activity substudy was begun in 2005 in a random sample of SWHS and SMHS participants; of 1,101 people contacted, 619 (56%) agreed to participate (Peters et al. 2010). The present analysis included 50 women and 50 men randomly sampled across strata of age and year of baseline urine collection from participants in this physical activity substudy. The study protocol was approved by institutional review boards of the participating institutions. The involvement of the CDC laboratory was determined not to constitute engagement in human subject research.

*Questionnaires*. During the initial contact at cohort enrollment, SWHS participants were given a self-administered questionnaire that elicited information on demographics, lifestyle, medical history, residence, and occupation. SWHS participants then completed an in-person, in-home interview focused on diet, reproductive history, physical activity, and water consumption either at the initial contact or within 2–3 days of initial contact. SMHS participants similarly completed in-person interviews on sociodemographic, lifestyle, physical activity, diet, and chronic health conditions. Brief interviews at the time of each urine sample collection elicited information about recent diet and smoking; information about medication use within the previous 24 hr was obtained at the first (baseline) collection. Weight was measured at baseline and approximately midway between collection of the second and third urine samples.

*Urine samples*. Spot urine samples were collected from 65,754 (88%) SWHS and 54,668 (89%) SMHS cohort members at the baseline interview. Baseline urine samples in the present study were collected primarily in 1998–1999 for the women (92%) and in 2003–2004 for the men (100%). A second and third spot urine sample was collected from physical activity substudy participants during two additional home interviews; 535 (86%) of these participants provided biological samples, and 516 (96%) of those providing biological samples did so at both interviews. Demographic, anthropometric, and lifestyle characteristics of cohort members who participated in the substudy and provided biological samples were similar to those of the full cohorts, suggesting that selection bias related to participating and providing urine samples was unlikely. The second sample was collected in 2006, an average of 6.7 years and 2.2 years after the baseline sample, for women and men, respectively. The third sample was collected approximately 9 months after the second. Urine was collected into sterilized 100 mL polypropylene cups containing 125 mg ascorbic acid, kept cold with ice packs (0–4°C) until processing within 6 hr of collection, and then stored at –70 to –80°C. Approximately 33% of the baseline urine samples was collected in the morning, with the rest collected in the afternoon or evening (Table 1). Almost all of the second and third urine samples (94% and 97%, respectively) were collected in the morning.

**Table 1:** Characteristics at enrollment of participants randomly selected from the SWHS and SMHS.

Characteristic	Total (*n* = 100)	Women (*n* = 50)	Men (*n* = 50)
Age at enrollment (years)
40–49	40 (40)	27 (54)	13 (26)
50–59	33 (33)	13 (26)	20 (40)
60–72	27 (27)	10 (20)	17 (34)
Highest educational level attained
Elementary school or no formal education	16 (16)	11 (22)	5 (10)
Middle school	38 (38)	19 (38)	19 (38)
High school	30 (30)	13 (26)	17 (34)
≥ Technical school/college	16 (16)	7 (14)	9 (18)
BMI (kg/m^2^)
< 20	6 (6)	4 (8)	2 (4)
20–24.9	55 (55)	25 (50)	30 (60)
25–29.9	34 (34)	17 (34)	17 (34)
≥ 30	5 (5)	4 (8)	1 (2)
Menopausal status
Premenopausal		26 (52)
Postmenopausal		23 (46)
Unknown		1 (2)
Cigarette smoking status
Current	33 (33)	2 (4)	31 (62)
Former	4 (4)	0 (0)	4 (8)
Never	63 (63)	48 (96)	15 (30)
No. of cigarettes smoked/day (among current smokers)
1–6	3 (9)	2 (100)	1 (3)
7–12	11 (33)	0 (0)	11 (35)
≥ 13	19 (58)	0 (0)	19 (61)
Family income in previous year^*a*^
Low	65 (65)	30 (60)	35 (70)
High	35 (35)	20 (40)	15 (30)
Usual source of drinking water
Tap water only		33 (66)
Tap water and bottled water		17 (34)
Any medicine taken within previous 24 hr at baseline
Yes	59 (59)	28 (56)	31 (62)
No	41 (41)	22 (44)	19 (38)
Time of day of collection for baseline urine sample (hours)
0700–0859	4 (4)	3 (6)	1 (2)
0900–1159	29 (29)	12 (24)	17 (34)
1200–1459	12 (12)	6 (12)	6 (12)
1500–1959	55 (55)	29 (58)	26 (52)
Time of day of collection for second urine sample
0700–0859	71 (71)	38 (76)	33 (66)
0900–1159	23 (23)	8 (16)	15 (30)
1200–1459	4 (4)	2 (4)	2 (4)
1500–1959	2 (2)	2 (4)	0 (0)
Time of day of collection for third urine sample
0700–0859	66 (66)	32 (64)	34 (68)
0900–1159	31 (31)	17 (34)	14 (28)
1200–1459	2 (2)	0 (0)	2 (4)
1500–1959	1 (1)	1 (2)	0 (0)
Values shown are n (%). ^***a***^“Low” and “high” annual income defined using cut points of 20,000 yuan for women and 12,000 yuan for men, which represent the approximate medians reported by each sex.			

*Laboratory analyses*. For each participant, one 750-μL aliquot was obtained from each of three banked urine samples. Participant and blind quality control (QC) samples were sent on dry ice by overnight mail to the CDC laboratory in Atlanta, Georgia, where 2,4-dichlorophenol, 2,5-dichlorophenol, butyl paraben, methyl paraben, propyl paraben, benzophenone-3, BPA, and triclosan were measured using previously published laboratory and QC methods (Ye et al. 2005, 2006). The limits of detection (LODs) were 0.2 μg/L (2,4-dichlorophenol, 2,5-dichlorophenol, butyl paraben, propyl paraben), 0.4 μg/L (benzophenone-3, BPA), 1.0 μg/L (methyl paraben), and 2.3 μg/L (triclosan). Urine samples for each three-sample set were analyzed in the same batch to minimize the impact of interbatch variability. Results from a blind QC pool indicated excellent reliability for most analytes, with interbatch coefficients of variation (CVs) < 0.10; reliability was lower for BPA [CV = 0.18, geometric mean (GM) = 1.8 μg/L], triclosan (CV = 0.22, GM = 5.9 μg/L), and 2,4-dichlorophenol (CV = 0.26, GM = 1.0 μg/L). As expected, CVs were higher for analytes at low concentrations.

*Statistical analyses*. We present analyte concentrations both creatinine-corrected (in micrograms per gram creatinine) and uncorrected (in micrograms per liter of urine) because age and chronic health conditions associated with age, such as chronic kidney disease, can influence creatinine clearance and, thus also influence the impact of using creatinine to adjust for urinary dilution (Waikar et al. 2010). Statistical analyses were performed only for analytes with detectable concentrations in ≥ 50% of samples (2,4-dichlorophenol, 2,5-dichlorophenol, BPA, methyl paraben, propyl paraben) (Table 2). Samples with creatinine concentrations < 20 mg/dL (two men’s baseline samples and one woman’s second sample) or > 275 mg/dL (one woman’s baseline sample) were excluded from analysis. Statistical analyses were conducted separately for males and females.

**Table 2:** Urinary phenol concentrations in the SWHS and SMHS.^*a*^

Phenol	Baseline		Sample 2		Sample 3	
	Percent > LOD^*b*^	GM (5th, 95th percentile)	Percent > LOD^*b*^	GM (5th, 95th percentile)	Percent > LOD^*b*^	GM (5th, 95th percentile)
Women
Uncorrected (μg/L)
2,4-Dichloro­phenol	89.8	1.29 (< LOD, 19.4)	89.8	1.06 (< LOD, 33.0)	94.0	1.31 (< LOD, 7.40)
2,5-Dichloro­phenol	100.0	32.1 (2.90, 655)	100.0	37.2 (4.20, 983)	98.0	30.7 (3.40, 368)
Benzophenone-3	28.6	NA (< LOD, 3.10)	38.8	NA (< LOD, 40.6)	40.0	NA (< LOD, 4.30)
BPA	65.3	0.622 (< LOD, 2.30)	65.3	0.661 (< LOD, 2.10)	80.0	0.888 (< LOD, 3.80)
Butyl paraben	22.5	NA (< LOD, 5.20)	30.6	NA (< LOD, 8.80)	44.0	NA (< LOD, 40.4)
Methyl paraben	98.0	21.1 (1.90, 151)	100.0	21.5 (2.20, 209)	96.0	28.8 (1.00, 289)
Propyl paraben	71.4	2.54 (< LOD, 50.6)	79.6	2.07 (< LOD, 90.0)	82.0	3.06 (< LOD, 103)
Triclosan	32.7	NA (< LOD, 112)	32.7	NA (< LOD, 36.7)	46.0	NA (< LOD, 232)
Creatinine- corrected (μg/g)
2,4-Dichloro­phenol	89.8	1.93 (< LOD, 18.0)	89.8	1.76 (< LOD, 45.6)	94.0	1.88 (< LOD, 11.9)
2,5-Dichloro­phenol	100.0	48.1 (4.00, 678)	100.0	61.6 (9.87, 1477)	98.0	44.1 (5.58, 689)
Benzophenone-3	28.6	NA (< LOD, 3.84)	38.8	NA (< LOD, 27.7)	40.0	NA (< LOD, 8.40)
BPA	65.3	0.931 (< LOD, 3.98)	65.3	1.10 (< LOD, 4.21)	80.0	1.27 (< LOD, 3.45)
Butyl paraben	22.5	NA (< LOD, 7.29)	30.6	NA (< LOD, 25.2)	44.0	NA (< LOD, 24.9)
Methyl paraben	98.0	31.6 (3.65, 258)	100.0	35.6 (3.15, 262)	96.0	41.3 (2.73, 355)
Propyl paraben	71.4	3.79 (< LOD, 137)	79.6	3.42 (< LOD, 100)	82.0	4.38 (< LOD, 101)
Triclosan	32.7	NA (< LOD, 134)	32.7	NA (< LOD, 77.8)	46.0	NA (< LOD, 244)
Men
Uncorrected (μg/L)
2,4-Dichloro­phenol	87.5	1.12 (< LOD, 18.1)	90.0	1.06 (< LOD, 8.70)	96.0	1.50 (0.300, 16.7)
2,5-Dichloro­phenol	100.0	37.5 (3.30, 730)	100.0	30.9 (6.70, 333)	100.0	47.5 (5.20, 855)
Benzophenone-3	25.0	NA (< LOD, 3.40)	30.0	NA (< LOD, 2.10)	26.0	NA (< LOD, 1.50)
BPA	75.0	0.683 (< LOD, 2.20)	74.0	0.774 (< LOD, 3.40)	86.0	0.794 (< LOD, 1.90)
Butyl paraben	14.6	NA (< LOD, 0.500)	22.0	NA (< LOD, 3.00)	20.0	NA (< LOD, 2.40)
Methyl paraben	97.9	9.31 (1.50, 94.3)	94.0	15.7 (< LOD, 119)	98.0	12.8 (1.20, 159)
Propyl paraben	54.2	0.450 (< LOD, 27.6)	70.0	1.06 (< LOD, 19.6)	64.0	0.843 (< LOD, 37.2)
Triclosan	27.1	NA (< LOD, 109)	24.0	NA (< LOD, 63.6)	24.0	NA (< LOD, 78.3)
Creatinine-corrected (μg/g)
2,4-Dichloro­phenol	87.5	1.18 (< LOD, 20.7)	90.0	1.09 (< LOD, 13.2)	96.0	1.48 (0.300, 12.9)
2,5-Dichloro­phenol	100.0	39.5 (8.41, 761)	100.0	31.9 (6.44, 526)	100.0	46.9 (7.65, 561)
Benzophenone-3	25.0	NA (< LOD, 2.44)	30.0	NA (< LOD, 2.70)	26.0	NA (< LOD, 1.78)
BPA	75.0	0.720 (< LOD, 1.60)	74.0	0.800 (< LOD, 3.42)	86.0	0.784 (< LOD, 1.67)
Butyl paraben	14.6	NA (< LOD, 0.450)	22.0	NA (< LOD, 3.78)	20.0	NA (< LOD, 1.66)
Methyl paraben	97.9	9.81 (1.90, 65.6)	94.0	16.2 (< LOD, 175)	98.0	12.6 (1.14, 149)
Propyl paraben	54.2	0.474 (< LOD, 18.7)	70.0	1.10 (< LOD, 17.3)	64.0	0.833 (< LOD, 40.3)
Triclosan	27.1	NA (< LOD, 97.0)	24.0	NA (< LOD, 64.8)	24.0	NA (< LOD, 46.8)
NA, not available; GM was not calculated if > 50% of samples were below LOD. Samples with creatinine < 20 or > 275 mg/dL were excluded. Baseline samples collected during 1998–2002 for women and 2003–2004 for men; second samples collected during 2006; third samples collected during 2006–2007. ^***a***^Number of samples: for women, *n*_baseline_ = 49, *n*_sample 2_ = 49, *n*_sample 3_ = 50; for men, *n*_baseline_ = 48, *n*_sample 2_ = 50, *n*_sample 3_ = 50. ^***b***^LODs are 2,4-dichloro­phenol, 0.2 μg/L; 2,5-dichloro­phenol, 0.2 μg/L; benzophenone-3, 0.4 μg/L; BPA, 0.4 μg/L; butyl paraben, 0.2 μg/L; methyl paraben, 1.0 μg/L; propyl paraben, 0.2 μg/L; triclosan, 2.3 μg/L.						

Values below the LOD were replaced by the LOD divided by the square root of 2 for all analyses (Hornung and Reed 1990). Because concentrations were log distributed, values were log-transformed for tests that required a normal distribution. GMs and 95% confidence intervals (CIs) were calculated. The Satterthwaite *t*-test was used to investigate binary predictors of concentration. Dose–response relations were evaluated using least squares regression of log-transformed concentrations on continuous measures of age, education, income, and body mass index (BMI). We also used multiple linear regression to simultaneously examine multiple predictors of log-transformed creatinine-corrected concentrations of individual phenols. To screen the large number of potential predictors and obtain parsimonious models, we included only predictors with *p* < 0.1 in univariate analyes for a given phenol and limited analyses to phenols with two or more such predictors. Concentrations were compared with those from the U.S. National Health and Nutrition Examination Surveys (NHANES), using the corresponding NHANES results nearest the sampling window of the Shanghai samples.

Intraindividual variability of phenol concentrations over time was evaluated in two ways. For each analyte, intraclass correlation coefficients (ICCs) and 95% CIs of log-transformed baseline, sample 2, and sample 3 values were estimated using mixed effects models with batch and participant included as random effects. The ICC was calculated as σ*_B_*^2^/(σ*_W_*^2^ + σ*_B_*^2^) [between-participant variance/(within-participant variance + between-participant variance)]. The association between continuous concentrations at baseline and the average of samples 2 and 3 was evaluated using the nonparametric Spearman’s rank correlation coefficient (SCC). The average of samples 2 and 3 was used for calculating the SCC because these samples were collected relatively close in time compared with the time between samples 1 and 2, and their average was considered more likely to provide a better estimate of an individual’s usual exposure in this later period than either sample individually. Analyses were performed using SAS (version 9.2; SAS Institute Inc., Cary, NC, USA). Statistical significance was assessed at the 5% level.

## Results

The study participants were similar to their respective parent cohorts (Cai et al. 2007; Zheng et al. 2005) across a range of demographic and lifestyle factors (Table 1). Mean age at enrollment was 50.5 years (women) and 56.3 years (men). None of the women were pregnant at enrollment. A substantially higher proportion of men than women were current cigarette smokers. Usual source of drinking water, which was ascertained only for women, was tap water among 66% of participants.

Most participants had detectable baseline urine concentrations of 2,4-dichlorophenol, 2,5-dichlorophenol, BPA, methyl paraben, and propyl paraben, ranging from 71.4% of women and 54.2% of men for propyl paraben to 100% of women and men for 2,5-dichlorophenol (Table 2). Benzophenone-3, butyl paraben, and triclosan were detected in < 50% of baseline samples for both sexes. The proportion of detectable concentrations remained similar or increased for all analytes between the baseline and third samples, although the second samples suggested possible nonmonotonicity for some analytes; the increases were appreciable for benzophenone-3, BPA, butyl paraben, propyl paraben, and triclosan in at least one sex. Benzophenone-3, butyl paraben, propyl paraben, and triclosan were consistently detected more often among women, whereas BPA was consistently detected more frequently among men. As expected, creatinine concentrations were appreciably higher among men (data not shown).

Although trends were not monotonic for all analytes, mean creatinine-corrected concentrations of BPA, methyl paraben, and propyl paraben increased modestly between the baseline and third samples for both sexes, whereas 2,4-dichlorophenol and 2,5-dichlorophenol concentrations increased only among men and were similar or decreased slightly among women (Table 2). The magnitude of increase differed by analyte and sex: BPA increased 36.4% among women and 8.9% among men, methyl paraben increased 28.4–30.7% in women and men; propyl paraben, with the greatest relative difference between the sexes, increased 15.6% among women and 75.7% among men. The pattern was similar for creatinine-corrected and uncorrected concentrations.

Creatinine-corrected concentrations of 2,4-dichlorophenol and 2,5-dichlorophenol were higher in the baseline samples compared with the 2003–2004 NHANES, whereas the opposite was seen for BPA, methyl paraben, and propyl paraben (see Supplemental Material, Table S1). This pattern was consistent for both sexes and remained unchanged for the third sample compared with the 2007–2008 NHANES. There was a general pattern of increasing concentrations over time in the Shanghai samples, whereas the pattern was more mixed in NHANES. The uncorrected measurements showed similar patterns (see Supplemental Material, Table S2).

Table 3 shows GMs of creatinine-corrected concentrations at baseline, stratified by selected factors. GMs of methyl paraben and propyl paraben were significantly higher among women. The GM of BPA also appeared higher among women (*p* = 0.06), despite the somewhat higher prevalence of detection and uncorrected concentrations of BPA among men (see Supplemental Material, Table S3). Income showed significant dose–response trends with 2,4-dichlorophenol, 2,5-dichlorophenol, and BPA concentrations, although no associations were observed in dichotomous analyses. Propyl paraben GMs were significantly lower among current smokers than among former or never-smokers. Women who drank primarily bottled water had significantly higher urinary concentrations of 2,4-dichlorophenol and 2,5-dichlorophenol and appeared to have higher concentrations of BPA (*p* = 0.08) compared with women who drank primarily tap water. Use of medication within the previous 24 hr was associated with significantly higher urinary concentrations of propyl paraben. BPA concentration was significantly higher in urine collected in the afternoon/evening compared with urine collected in the morning.

**Table 3:** GMs (95% CIs) of creatinine-corrected baseline sample urinary phenol concentrations (μg/g) by selected characteristics.

Characteristic	*n*	2,4-Dichloro­phenol	2,5-Dichloro­phenol	BPA	Methyl paraben	Propyl paraben
Sex
Female	49	1.93 (1.30, 2.88)	48.1 (30.4, 76.0)	0.931 (0.754, 1.15)	31.6 (21.1, 47.2)	3.79 (1.82, 7.92)
Male	48	1.18 (0.741, 1.87)	39.5 (25.3, 61.6)	0.720 (0.608, 0.851)	9.81 (7.02, 13.7)	0.474 (0.281, 0.797)
*p*-Value^*a*^		0.10	0.54	0.06	< 0.001	< 0.001
Age (years)
≤ 50	42	1.69 (1.04, 2.75)	57.4 (35.1, 94.0)	0.811 (0.636, 1.04)	19.6 (12.9, 29.9)	1.50 (0.726, 3.09)
> 50	55	1.39 (0.937, 2.06)	35.3 (23.4, 53.4)	0.826 (0.706, 0.966)	16.4 (11.1, 24.2)	1.25 (0.630, 2.50)
*p*-Value^*a*^		0.53	0.13	0.90	0.52	0.72
Trend* p-*value^*b*^		0.77	0.48	0.91	0.04	0.31
Education
≤ Middle school	52	1.50 (1.00, 2.23)	41.3 (27.54, 61.8)	0.813 (0.677, 0.977)	15.3 (10.3, 22.8)	1.34 (0.687, 2.62)
> Middle school	45	1.53 (0.948, 2.47)	46.4 (27.9, 77.3)	0.826 (0.671, 1.02)	20.9 (13.8, 31.6)	1.37 (0.644, 2.91)
*p*-Value^*a*^		0.94	0.71	0.91	0.28	0.97
Trend* p-*value^*b*^		0.45	0.21	0.71	0.22	0.76
Income^*c*^
Low	62	1.33 (0.912, 1.95)	40.1 (27.4, 58.7)	0.768 (0.655, 0.902)	14.9 (10.4, 21.3)	1.08 (0.581, 2.02)
High	35	1.89 (1.13, 3.17)	50.6 (28.5, 89.9)	0.918 (0.714, 1.18)	24.0 (15.0, 38.2)	2.02 (0.881, 4.61)
*p*-Value^*a*^		0.28	0.50	0.23	0.11	0.23
Trend* p-*value^*b*^		0.02	0.02	0.05	0.19	0.22
Baseline BMI (kg/m^2^)
< 25	59	1.58 (1.06, 2.37)	45.7 (30.4, 68.7)	0.871 (0.726, 1.04)	17.0 (11.6, 24.8)	1.15 (0.610, 2.18)
≥ 25	38	1.41 (0.871, 2.27)	40.5 (24.1, 68.1)	0.746 (0.607, 0.916)	18.9 (12.1, 29.4)	1.74 (0.779, 3.88)
*p*-Value^*a*^		0.70	0.72	0.26	0.71	0.42
Trend* p-*value^*b*^		0.25	0.36	0.74	0.73	0.95
Smoking^*d*^
Former/never	19	1.22 (0.516, 2.89)	37.5 (15.7, 89.7)	0.709 (0.557, 0.904)	13.8 (7.43, 25.8)	0.951 (0.364, 2.49)
Current	29	1.15 (0.654, 2.01)	40.8 (24.3, 68.4)	0.726 (0.571, 0.924)	7.83 (5.33, 11.5)	0.300 (0.168, 0.534)
*p*-Value^*a*^		0.90	0.86	0.89	0.11	0.04
≥ 13 Cigarettes/day among smokers^*d*^
No	12	0.702 (0.259, 1.91)	29.2 (13.4, 63.9)	0.801 (0.514, 1.25)	7.33 (3.46, 15.6)	0.350 (0.130, 0.944)
Yes	17	1.62 (0.811, 3.24)	51.6 (24.7, 108.)	0.678 (0.499, 0.921)	8.19 (5.14, 13.1)	0.269 (0.123, 0.588)
*p*-Value^*a*^		0.15	0.26	0.51	0.79	0.66
Consume bottled water^*e*^
No	32	1.32 (0.844, 2.05)	30.7 (18.4, 51.2)	0.792 (0.661, 0.951)	35.7 (20.8, 61.4)	4.55 (1.73, 12.0)
Yes	17	4.00 (1.95, 8.21)	112.0 (48.6, 256.)	1.26 (0.758, 2.09)	25.0 (13.6, 46.0)	2.70 (0.799, 9.11)
*p*-Value^*a*^		0.01	0.01	0.08	0.37	0.49
Postmenopausal^*e*^
No	25	2.09 (1.16, 3.75)	52.0 (26.1, 104.)	0.980 (0.686, 1.40)	33.8 (18.9, 60.5)	5.61 (2.27, 13.9)
Yes	23	1.68 (0.931, 3.03)	41.1 (21.1, 79.8)	0.888 (0.685, 1.15)	27.5 (14.9, 50.5)	2.16 (0.629, 7.44)
*p*-Value^*a*^		0.59	0.61	0.65	0.61	0.21
Took medicine within previous 24 hr
No	39	1.43 (0.902, 2.27)	41.9 (25.9, 67.8)	0.841 (0.677, 1.05)	14.8 (10.4, 21.0)	0.649 (0.352, 1.20)
Yes	58	1.57 (1.04, 2.37)	44.8 (29.2, 68.6)	0.805 (0.674, 0.962)	20.0 (13.2, 30.3)	2.22 (1.11, 4.46)
*p*-Value^*a*^		0.77	0.83	0.75	0.27	0.009
Time of day of urine collection
Morning	31	1.52 (0.889, 2.59)	48.4 (29.6, 79.2)	0.639 (0.508, 0.804)	16.3 (9.52, 27.8)	0.930 (0.371, 2.33)
Afternoon/evening	66	1.51 (1.04, 2.20)	41.5 (27.6, 62.4)	0.921 (0.782, 1.08)	18.4 (13.1, 25.9)	1.62 (0.894, 2.92)
*p*-Value^*a*^		0.99	0.63	0.01	0.69	0.31
Samples with creatinine < 20 or > 275 mg/dL were excluded. Benzophenone-3, butyl paraben, and triclosan were excluded because > 50% of samples were below LOD. ^***a***^*p*-Value for Satterthwaite *t*-test. ^***b***^*p*-Value for continuous measure in linear regression models testing dose response (age, education, income, and BMI only). ^***c***^High income was defined as ≥ 20,000 yuan/year for females and ≥ 12,000 yuan/year for males. ^***d***^Males only. ^***e***^Females only.						

Creatinine-corrected concentrations in the third sample were also significantly higher among women for BPA, methyl paraben, and propyl paraben (Table 4). Propyl paraben concentrations appeared to be higher among participants ≤ 50 years of age compared with older participants (*p* = 0.06). Income showed positive associations with 2,4-dichlorophenol, 2,5-dichlorophenol, and BPA concentrations in dichotomous analyses (*p* < 0.06) and significant dose–response trends. Concentrations of methyl paraben were lower among current smokers compared with former or never-smokers. Similar patterns were observed for uncorrected concentrations (see Supplemental Material, Table S4).

**Table 4:** GMs (95% CIs) of creatinine-corrected third sample urinary phenol concentrations (μg/g) by selected characteristics.

Characteristic	*n*	2,4-Dichloro­phenol	2,5-Dichloro­phenol	BPA	Methyl paraben	Propyl paraben
Sex
Female	50	1.88 (1.34, 2.63)	44.1 (27.7, 70.2)	1.27 (1.05, 1.54)	41.3 (27.6, 61.7)	4.38 (2.25, 8.55)
Male	50	1.48 (1.05, 2.09)	46.9 (31.6, 69.7)	0.784 (0.673, 0.914)	12.6 (8.24, 19.3)	0.833 (0.450, 1.54)
*p*-Value^*a*^		0.32	0.84	< 0.001	< 0.001	< 0.001
Age (years)
≤ 50	44	1.83 (1.23, 2.71)	49.9 (30.2, 82.6)	1.00 (0.830, 1.20)	26.7 (15.6, 45.8)	3.20 (1.49, 6.86)
> 50	56	1.55 (1.15, 2.10)	42.2 (29.0, 61.5)	1.00 (0.830, 1.20)	20.2 (13.9, 29.3)	1.28 (0.698, 2.33)
*p*-Value^*a*^		0.52	0.59	1.00	0.39	0.06
Trend* p-*value^*b*^		0.59	0.77	0.59	0.44	0.34
Education
≤ Middle school	54	1.64 (1.17, 2.28)	45.5 (30.1, 68.8)	1.02 (0.843, 1.23)	23.2 (15.2, 35.3)	2.06 (1.07, 3.98)
> Middle school	46	1.71 (1.20, 2.43)	45.4 (28.9, 71.4)	0.980 (0.815, 1.18)	22.4 (13.9, 36.2)	1.75 (0.857, 3.57)
*p*-Value^*a*^		0.86	0.99	0.78	0.92	0.74
Trend* p-*value^*b*^		0.68	0.53	0.53	0.84	0.87
Income^c^
Low	65	1.43 (1.04, 1.97)	37.4 (25.0, 55.8)	0.908 (0.775, 1.06)	20.6 (13.6, 31.3)	1.48 (0.802, 2.72)
High	35	2.23 (1.60, 3.09)	65.5 (43.0, 99.7)	1.20 (0.955, 1.50)	27.6 (17.5, 43.6)	3.08 (1.43, 6.63)
*p*-Value^*a*^		0.05	0.06	0.05	0.34	0.13
Trend* p-*value^*b*^		0.03	0.04	0.03	0.21	0.10
BMI
< 25 kg/m^2^	56	1.61 (1.18, 2.19)	42.3 (28.3, 63.2)	0.997 (0.854, 1.16)	25.1 (16.2, 38.9)	2.29 (1.17, 4.47)
≥ 25 kg/m^2^	41	1.73 (1.14, 2.62)	50.8 (30.8, 83.9)	1.01 (0.798, 1.28)	20.4 (12.6, 32.8)	1.54 (0.747, 3.18)
*p*-Value^*a*^		0.78	0.57	0.93	0.52	0.42
Trend* p-*value^*b*^		0.91	0.98	0.90	0.53	0.78
Smoking^*d*^
Former/never	19	1.70 (0.852, 3.37)	53.3 (27.1, 105.)	0.764 (0.584, 1.00)	24.6 (14.0, 43.4)	1.60 (0.509, 5.01)
Current	31	1.36 (0.912, 2.03)	43.4 (26.0, 72.4)	0.797 (0.655, 0.970)	8.38 (4.75, 14.8)	0.559 (0.272, 1.15)
*p*-Value^*a*^		0.57	0.62	0.79	0.008	0.12
≥13 Cigarettes/day among smokers^*d*^
No	12	1.38 (0.648, 2.93)	49.9 (20.4, 122.)	0.831 (0.632, 1.09)	7.17 (2.96, 17.4)	0.513 (0.171, 1.54)
Yes	19	1.35 (0.811, 2.25)	39.7 (20.0, 78.8)	0.777 (0.582, 1.04)	9.24 (4.15, 20.6)	0.590 (0.210, 1.66)
*p*-Value^*a*^		0.96	0.66	0.72	0.65	0.84
Consume bottled water^*e*^
No	33	1.87 (1.21, 2.88)	42.6 (22.7, 79.6)	1.26 (0.985, 1.60)	33.3 (19.7, 56.1)	3.90 (1.67, 9.08)
Yes	17	1.91 (1.07, 3.41)	47.1 (23.0, 96.6)	1.31 (0.926, 1.86)	62.7 (33.3, 118.)	5.51 (1.66, 18.3)
*p*-Value^*a*^		0.95	0.82	0.83	0.12	0.63
Postmenopausal^*e*^
No	26	2.24 (1.39, 3.60)	47.8 (23.2, 98.3)	1.22 (0.972, 1.54)	50.7 (29.9, 86.0)	7.42 (2.97, 18.5)
Yes	23	1.50 (0.898, 2.51)	37.5 (19.8, 71.0)	1.27 (0.912, 1.77)	34.4 (17.7, 67.0)	2.57 (0.909, 7.24)
*p*-Value^*a*^		0.25	0.61	0.86	0.35	0.12
Samples with creatinine < 20 or > 275 mg/dL were excluded. ^***a***^*p*-Value for Satterthwaite t-test. ^***b***^*p*-Value for continuous measure in linear regression models testing dose response (age, education, income, and BMI only). ^***c***^High income was defined as ≥ 20,000 yuan/year for females and ≥ 12,000 yuan/year for males. ^***d***^Males only. ^***e***^Females only.						

In multivariate models for individual analytes, the above predictors of creatinine-corrected concentrations generally remained significant, except for age, in years, in relation to methyl paraben in the first sample and > 50 years of age in relation to propyl paraben in the third sample (Table 5).

**Table 5:** Univariate and multiple linear regression coefficients (95% CI) for creatinine-corrected urinary phenol concentrations and selected participant/specimen characteristics, by specimen collection.

Phenol and characteristic	Regression coefficient	
	Univariate models	Multivariate models
Baseline sample
BPA
Male (vs. female)	–0.26 (–0.52, 0.01)	–0.12 (–0.40, 0.16)
Annual income (per 1,000 yuan, continuous)	0.21 (0.05, 0.36)	0.17 (0.01, 0.34)
Urine collected in afternoon/evening (vs. morning)	0.36 (0.09, 0.64)	0.35 (0.08, 0.62)
Methyl paraben
Male (vs. female)	–1.17 (–1.68, –0.66)	–1.09 (–1.63, –0.55)
Age (per year, continuous)	–0.03 (–0.06, 0.00)	–0.01 (–0.04, 0.02)
Propyl paraben
Male (vs. female)	–2.08 (–2.96, –1.20)	–2.15 (–3.00, –1.31)
Took medicine in previous 24 hr (vs. no medicine in previous 24 hr)	1.23 (0.26, 2.19)	1.35 (0.49, 2.21)
Third sample
BPA
Male (vs. female)	–0.48 (–0.72, –0.25)	–0.37 (–0.63, –0.12)
Annual income (per 1,000 yuan, continuous)	0.26 (0.11, 0.41)	0.17 (0.02, 0.33)
Propyl paraben
Male (vs. female)	–1.66 (–2.55, –0.77)	–1.54 (–2.44, –0.64)
Age > 50 years (vs. ≤ 50 years)	–0.92 (–1.85, 0.01)	–0.61 (–1.52, 0.30)
Samples with creatinine < 20 or > 275 mg/dL were excluded. Multiple linear regression models for each phenol include all predictors that were statistically significant at *p* < 0.1 in univariate analyses. Certain models could not be tested because a given predictor was defined for only a subgroup (e.g., bottled water consumption) or because of collinearity (e.g., sex and cigarette smoking).		

Temporal reproducibility among the three samples per participant tended to be higher for men than for women (Table 6). We observed moderately high ICCs and SCCs among men for 2,4-dichlorophenol and 2,5-dichlorophenol, with values ranging from 0.54 to 0.60 (ICC) and 0.43 to 0.56 (SCC). The corresponding measures among women were 0.29–0.39 and 0.12–0.35, respectively, indicating poor reproducibility. These measures were poor to fair for the other analytes. The ICCs and SCCs for 2,4-dichlorophenol and 2,5-dichlorophenol increased for both men and women when analyses were restricted to samples collected in the morning, whereas patterns for other analytes were inconsistent (see Supplemental Material, Table S5); however, CIs were very wide.

**Table 6:** Measures of reproducibility for urinary phenol concentrations.

Phenol	Females		Males	
	ICC (95% CI)^*a*^	Correlation^*b*^	ICC (95% CI)^*a*^	Correlation^*b*^
Uncorrected (μg/L)
2,4-Dichloro­phenol	0.35 (0.21, 0.51)	0.22	0.55 (0.43, 0.67)	0.55
2,5-Dichloro­phenol	0.29 (0.16, 0.47)	0.12	0.59 (0.47, 0.70)	0.52
BPA	0.07 (0.01, 0.50)	0.13	0.32 (0.19, 0.49)	0.31
Methyl paraben	0.24 (0.12, 0.43)	0.44	0.22 (0.10, 0.41)	0.24
Propyl paraben	0.31 (0.17, 0.48)	0.41	0.29 (0.16, 0.47)	0.36
Creatinine-corrected (μg/g)
2,4-Dichloro­phenol	0.39 (0.26, 0.54)	0.35	0.54 (0.41, 0.66)	0.43
2,5-Dichloro­phenol	0.32 (0.19, 0.49)	0.18	0.60 (0.48, 0.71)	0.56
BPA	0.13 (0.03, 0.40)	0.17	0.25 (0.13, 0.45)	0.04
Methyl paraben	0.21 (0.09, 0.41)	0.41	0.20 (0.08, 0.41)	0.17
Propyl paraben	0.30 (0.17, 0.47)	0.39	0.29 (0.16, 0.47)	0.35
Samples with creatinine < 20 or > 275 mg/dL were excluded. Number of samples: *n*_females_ = 49, *n*_males_ = 48. ^***a***^Baseline sample, sample 2, and sample 3. ^***b***^Baseline sample and the average of samples 2 and 3; values are Spearman correlation coefficients.				

## Discussion

These results suggest that, in this population of Shanghai adults, a single spot urine sample should allow a moderately reliable ranking of individuals’ exposure to the precursors of 2,4-dichlorophenol and 2,5-dichlorophenol relative to other participants among men and, under certain circumstances, among women. It is unlikely to be adequate for ranking of exposure to other phenols for either sex. We detected 2,4-dichlorophenol, 2,5-dichlorophenol, BPA, methyl paraben, and propyl paraben among most participants in the present study. Concentrations of several of the phenols, including methyl paraben, propyl paraben, BPA, and 2,4-dichlorophenol, were higher among women. Mean creatinine-corrected concentrations of BPA, methyl paraben, and propyl paraben increased over time for both sexes, whereas 2,4-dichlorophenol and 2,5-dichlorophenol concentrations increased only among men. Lifestyle factors related to urinary concentrations of one or more of these chemicals included smoking, consumption of bottled water, and income, although the relationships appeared to vary over the study period.

The mean BPA urinary concentrations observed in the present study are comparable to those reported in two Chinese studies among similarly aged persons during a similar time period (Ning et al. 2011; Zhang et al. 2011), although they are higher than those observed among persons > 40 years of age in a study of nonoccupationally exposed Chinese factory workers and their families (He et al. 2009). Studies in South Korea have reported urinary concentrations of BPA that are 2–3 times higher than those observed in the present study and in other Chinese studies (Kim et al. 2011; Zhang et al. 2011). Reasons for the observed differences are unclear.

The sex differences observed in the absolute concentrations and in the agreement over time in relative concentrations of some of these analytes are not surprising, given that several of the compounds, including the parabens, are present in cosmetics and other personal care products used primarily by women. It is noteworthy that most of these differences persisted across the time period of the present study.

The evidence for sex differences in body burdens of BPA is mixed, with some Asian studies reporting higher concentrations among men (He et al. 2009; Ning et al. 2011) and others reporting no difference (Kim et al. 2011; Li et al. 2013; Zhang et al. 2011). We found higher concentrations among women, which is consistent with NHANES (Calafat et al. 2008). The reason for this discrepancy is unclear. The association between smoking and BPA concentration is also uncertain, with one Chinese study reporting a positive association (He et al. 2009), but no association observed in other Asian studies (Kim et al. 2011; Ning et al. 2011), including the present study, nor in the United States (Calafat et al. 2008). The increased concentration of BPA that we observed in relation to consumption of bottled water, which approached statistical significance (*p* = 0.08), is consistent with the one other Chinese study that examined this (Li et al. 2013). The higher concentration of propyl paraben among persons who took medication within the previous 24 hr is intriguing, given that propyl paraben is a widely used preservative in pharmaceutical products (Soni et al. 2005). We have not seen this association reported in previous Chinese studies and we lacked the data necessary to investigate it further, including formulation and brand/source of the medications.

Data on triclosan exposure in Asia are limited. Studies among Chinese children and young adults (Li et al. 2013) and Korean adults (Kim et al. 2011) reported GMs of 3.77 μg/L and 1.68 μg/L, respectively. Most samples in our study were below the LOD of 2.3 μg/L.

Most studies of temporal variability of urinary BPA concentrations have been of short duration and have reported similarly low ICCs of around 0.1–0.2 (Braun et al. 2011, 2012; Meeker et al. 2011; Ye et al. 2011), although one study of children reported a somewhat higher ICC of 0.35 (Teitelbaum et al. 2008). A study of banked specimens collected from premenopausal women in the 1980s reported an SCC of 0.5 between samples collected 2 weeks apart, but 0.3 for samples collected 4 weeks apart (Nepomnaschy et al. 2009). Although one study reported moderate sensitivity of a single urine sample to predict mean BPA concentration tertiles over approximately 5 months (Mahalingaiah et al. 2008), most evidence suggests that a single measurement is unlikely to accurately reflect long-term exposure to BPA. In addition, Meeker et al (2011) reported SCCs and ICCs for creatinine-corrected concentrations of methyl paraben and propyl paraben among men that were comparable to those in the present study, although Smith et al (2012) observed somewhat higher ICCs for these chemicals. All but two of these studies (Nepomnaschy et al. 2009; Ye et al. 2011) used spot samples.

The degree to which a single urine sample reflects an individual’s long-term concentrations of these chemicals depends on the within-person variability of these concentrations over time. Greater within-person variability will result in increased measurement error of a single measurement and, consequently, greater attenuation of observed associations (e.g., relative risks). Understanding how well a single measurement reflects longer-term concentrations is essential for conducting and interpreting epidemiologic studies of these associations.

Study strengths include availability of serial urine samples collected over several years, allowing investigation of the reliability of exposure assessment over a substantially longer period than previously examined. We were also able to analyze this reliability for several phenols in addition to BPA and parabens. Our samples were analyzed in the same laboratory and with the same methods as NHANES samples, allowing direct comparison of measured concentrations between the United States and China. Last, we studied general population cohorts that were generally representative of middle-aged and older persons in Shanghai.

Limitations of the present study include its relatively small sample size, especially when stratified by sex. Nonetheless, we were able to identify a number of potential predictors of exposure in this population. Additional samples collected during follow-up would have allowed better estimation of exposure to these chemicals over time, although few long-term prospective cohorts have more than a baseline spot urine sample. Information on bottled water consumption, which was a potential predictor of exposure to several of the chemicals, was available only for women. In addition, we lacked detailed information on medications used. The small number of participants who provided second or third urine samples in the afternoon/evening limited our ability to account for timing of collection when estimating reliability. Last, some observed associations may be due to chance because of the large number of comparisons performed.

## Conclusions

Exposure to the precursors of 2,4-dichlorophenol and 2,5-dichlorophenol and to BPA, methyl paraben, and propyl paraben is widespread among Chinese adults > 40 years of age, with a smaller, but substantial and growing, proportion exposed to benzophenone-3, butyl paraben, and triclosan. Chinese women appear to be more exposed to these chemicals than do men, which may be due to differences in the use of personal care products such as cosmetics, antimicrobial soaps, and moisturizing lotions. Consumption of bottled water or related lifestyle factors may also be associated with greater exposure to several of these chemicals (or their precursors), including 2,4-dichlorophenol, 2,5-dichlorophenol, and BPA. The Chinese profile differs from that of the United States, with higher concentrations of 2,4-dichlorophenol and 2,5-dichlorophenol, but lower concentrations of BPA, methyl paraben, and propyl paraben. Lastly, our results suggest that, in similar populations, a single spot urine sample may be adequate for ranking exposure to the precursors of 2,4-dichlorophenol and 2,5-dichlorophenol among men and, under certain circumstances, among women, and that multiple measurements are needed to capture the exposure variability of the other chemicals. These findings require replication in other age and demographic groups but provide valuable information for future study of these chemicals in Chinese populations.

## Supplemental Material

(320 KB) PDF
