# Supplementary material for: Predictors and Variability of Repeat Measurements of Urinary Phenols and Parabens in a Cohort of Shanghai Women and Men
Source: Environ Health Perspect. 2014 Mar 21;122(7):733–40. doi: 10.1289/ehp.1306830 (PMC4080538; doi:10.1289/ehp.1306830)
Supplement: (320 KB) PDF [file ehp.1306830.s001.pdf]

Environmental Health Perspectives doi:10.1289/ehp.1306830

Supplemental Material

Predictors and Variability of Repeat Measurements of Urinary Phenols and Parabens in a Cohort of Shanghai Women and Men

Lawrence S. Engel, Jessie P. Buckley, Gong Yang, Linda M. Liao, Jaya Satagopan, Antonia M. Calafat, Charles E. Matthews, Qiuyin Cai, Bu-Tian Ji, Hui Cai, Stephanie M. Engel, Mary S. Wolff, Nathaniel Rothman, Wei Zheng, Yong-Bing Xiang, Xiao-Ou Shu, Yu-Tang Gao, and Wong-Ho Chow

Table of Contents **Page**

Table S1. Comparison of geometric means (95% CI) of creatinine-corrected urinary phenol concentrations (μg/g) between study participants and NHANES. **2**

Table S2. Comparison of geometric means (95% CI) of urinary phenol concentrations (μg/L) between study participants and NHANES. **3**

Table S3. Geometric means (95% CI) of baseline sample urinary phenol concentrations (μg/L) by selected characteristics. **4**

Table S4. Geometric means (95% CI) of third sample urinary phenol concentrations (μg/L) by selected characteristics. **6**

Table S5. Measures of reproducibility for urinary phenol concentrations, restricted to samples collected in the morning. **8**

Table S1. Comparison of geometric means (95% CI) of creatinine-corrected urinary phenol concentrations (μg/g) between study participants and NHANES.

Phenol	Total: current study	Total: NHANES	Females: current study	Females: NHANES	Males: current study	Males: NHANES
Baseline[a]						
2,4-dichlorophenol	1.51 (1.12, 2.05)	1.02 (0.873, 1.18)	1.93 (1.30, 2.88)	1.03 (0.845, 1.27)	1.18 (0.741, 1.86)	0.995 (0.850, 1.17)
2,5-dichlorophenol	43.6 (31.8, 59.7)	12.5 (10.1, 15.6)	48.1 (30.4, 76.0)	12.9 (9.91, 16.8)	39.5 (25.3, 61.6)	12.2 (9.73, 15.3)
Benzophenone-3	NA	22.2 (17.6, 28.0)	NA	35.5 (27.1, 46.4)	NA	13.6 (10.8, 17.1)
BPA	0.819 (0.716, 0.938)	2.58 (2.36, 2.82)	0.931 (0.754, 1.15)	2.78 (2.50, 3.08)	0.720 (0.608, 0.851)	2.38 (2.15, 2.63)
Butyl paraben	NA	NA	NA	1.06 (0.914, 1.24)	NA	NA
Methyl paraben	17.7 (13.3, 23.5)	55.0 (46.8, 64.6)	31.6 (21.1, 47.2)	123 (99.3, 152)	9.81 (7.02, 13.7)	23.9 (20.6, 27.8)
Propyl paraben	1.35 (0.828, 2.22)	7.71 (6.40, 9.30)	3.79 (1.82, 7.92)	23.9 (19.9, 28.8)	0.474 (0.281, 0.797)	2.38 (1.92, 2.95)
Triclosan	NA	12.7 (11.5, 14.1)	NA	12.2 (10.6, 14.2)	NA	13.2 (11.3, 15.6)
Sample 3[b]						
2,4-dichlorophenol	1.67 (1.31, 2.12)	0.978 (0.867, 1.10)	1.88 (1.34, 2.63)	1.07 (0.910, 1.26)	1.48 (1.05, 2.09)	0.891 (0.808, 0.984)
2,5-dichlorophenol	45.5 (33.7, 61.4)	9.12 (7.35, 11.3)	44.1 (27.7, 70.2)	9.07 (6.91, 11.9)	46.9 (31.6, 69.7)	9.17 (7.63, 11.0)
Benzophenone-3	NA	18.4 (13.9, 24.4)	NA	33.0 (25.0, 43.7)	NA	10.0 (7.37, 13.7)
BPA	1.00 (0.878, 1.14)	2.10 (1.95, 2.25)	1.27 (1.05, 1.54)	2.36 (2.17, 2.57)	0.784 (0.673, .914)	1.85 (1.71, 2.01)
Butyl paraben	NA	NA	NA	1.11 (0.984, 1.25)	NA	NA
Methyl paraben	22.8 (16.7, 31.2)	60.6 (49.8, 73.8)	41.3 (27.6, 61.7)	126 (109, 145)	12.6 (8.24, 19.3)	28.3 (22.8, 35.0)
Propyl paraben	1.91 (1.19, 3.08)	7.65 (6.22, 9.40)	4.38 (2.25, 8.55)	22.0 (18.7, 25.8)	0.833 (0.450, 1.54)	2.55 (2.06, 3.15)
Triclosan	NA	15.5 (13.7, 17.5)	NA	18.5 (15.6, 22.0)	NA	12.8 (10.9, 15.0)

Samples with creatinine < 20 or > 275 mg/dL were excluded.

NA: GM not calculated (>50% of samples below LOD).

[a]Baseline samples were collected during 1998-2002 for women and 2003-2004 for men (N_{total}=97, $N_{females}$=49, N_{males}=48). These are compared to NHANES 2003-2004 (N_{total}=2605, $N_{females}$=1355, N_{males}=1250), except for the parabens (NHANES 2005-2006; N_{total}=2548, $N_{females}$=1278, N_{males}=1270). [b]Third samples were collected during 2006-2007 (N_{total}=100, $N_{females}$=50, N_{males}=50). These are compared to NHANES 2007-2008 (N_{total}=2604, $N_{females}$=1310, N_{males}=1294).

Table S2. Comparison of geometric means (95% CI) of urinary phenol concentrations (µg/L) between study participants and NHANES.

Phenol	Total: current study	Total: NHANES	Females: current study	Females: NHANES	Males: current study	Males: NHANES
Baseline[a]						
2,4-dichlorophenol	1.20 (0.874, 1.65)	1.04 (0.895, 1.21)	1.29 (0.836, 2.00)	0.896 (0.754, 1.07)	1.12 (0.691, 1.80)	1.22 (1.02, 1.45)
2,5-dichlorophenol	34.7 (24.7, 48.8)	12.9 (10.1, 16.3)	32.1 (19.3, 53.5)	11.2 (8.51, 14.7)	37.5 (23.4, 60.0)	14.9 (11.8, 18.8)
Benzophenone-3	NA	22.9 (18.1, 28.9)	NA	30.7 (23.7, 39.8)	NA	16.8 (13.2, 21.3)
BPA	0.652 (0.567, 0.750)	2.64 (2.38, 2.94)	0.622 (0.511, 0.758)	2.41 (2.11, 2.75)	0.683 (0.556, 0.840)	2.92 (2.63, 3.24)
Butyl paraben	NA	NA	NA	0.904 (0.760, 1.07)	NA	NA
Methyl paraben	14.1 (10.7, 18.5)	56.4 (46.9, 67.9)	21.1 (14.4, 31.0)	104 (80.8, 135)	9.31 (6.44, 13.5)	29.8 (24.8, 35.8)
Propyl paraben	1.08 (0.672, 1.73)	7.91 (6.41, 9.77)	2.54 (1.25, 5.14)	20.4 (16.0, 25.9)	0.450 (0.261, 0.775)	2.96 (2.33, 3.77)
Triclosan	NA	13.0 (11.6, 14.6)	NA	10.6 (9.29, 12.1)	NA	16.2 (13.4, 19.6)
Sample 3[b]						
2,4-dichlorophenol	1.40 (1.07, 1.83)	0.970 (0.852, 1.11)	1.31 (0.916, 1.88)	0.893 (0.750, 1.06)	1.50 (1.00, 2.24)	1.06 (0.943, 1.19)
2,5-dichlorophenol	38.2 (27.5, 53.0)	9.04 (7.22, 11.3)	30.7 (19.1, 49.5)	7.57 (5.70, 10.1)	47.5 (30.1, 74.9)	10.9 (8.86, 13.4)
Benzophenone-3	NA	18.3 (13.7, 24.3)	NA	27.6 (20.4, 37.3)	NA	11.9 (8.84, 16.0)
BPA	0.840 (0.728, 0.969)	2.08 (1.92, 2.26)	0.888 (0.704, 1.12)	1.97 (1.80, 2.16)	0.794 (0.668, 0.944)	2.20 (2.01, 2.41)
Butyl paraben	NA	NA	NA	0.926 (0.825, 1.04)	NA	NA
Methyl paraben	19.2 (14.0, 26.2)	60.1 (49.6, 72.8)	28.8 (18.2, 45.6)	105 (90.2, 123)	12.8 (8.55, 19.1)	33.6 (27.3, 41.3)
Propyl paraben	1.61 (0.996, 2.59)	7.59 (6.22, 9.26)	3.06 (1.48, 6.31)	18.4 (15.7, 21.5)	0.843 (0.467, 1.52)	3.02 (2.47, 3.71)
Triclosan	NA	15.3 (13.5, 17.4)	NA	15.5 (12.6, 18.9)	NA	15.2 (12.9, 17.9)

Samples with creatinine < 20 or > 275 mg/dL were excluded.

NA: GM not calculated (>50% of samples below LOD).

[a]Baseline samples were collected during 1998-2002 for women and 2003-2004 for men (N_{total}=97, $N_{females}$=49, N_{males}=48). These are compared to NHANES 2003-2004 (N_{total}=2605, $N_{females}$=1355, N_{males}=1250), except for the parabens (NHANES 2005-2006; N_{total}=2548, $N_{females}$=1278, N_{males}=1270). [b]Third samples were collected during 2006-2007 (N_{total}=100, $N_{females}$=50, N_{males}=50). These are compared to NHANES 2007-2008 (N_{total}=2604, $N_{females}$=1310, N_{males}=1294).

Table S3. Geometric means (95% CI) of baseline sample urinary phenol concentrations (μg/L) by selected characteristics.

**Characteristic**	**N**	**2,4-dichlorophenol**	**2,5-dichlorophenol**	**BPA**	**Methyl paraben**	**Propyl paraben**
Sex						
Female	49	1.29 (0.836, 2.00)	32.1 (19.3, 53.5)	0.622 (0.511, 0.758)	21.1 (14.4, 31.0)	2.54 (1.25, 5.14)
Male	48	1.12 (0.691, 1.80)	37.5 (23.4, 60.0)	0.683 (0.556, 0.840)	9.31 (6.44, 13.5)	0.450 (0.261, 0.775)
p-value[a]		0.65	0.66	0.51	0.003	< 0.001
Age (years)						
≤50	42	1.32 (0.823, 2.13)	45.0 (27.8, 72.8)	0.636 (0.512, 0.789)	15.4 (9.88, 24.0)	1.17 (0.561, 2.45)
>50	55	1.12 (0.719, 1.74)	28.4 (17.5, 46.1)	0.665 (0.55, 0.804)	13.2 (9.21, 18.8)	1.01 (0.535, 1.91)
p-value[a]		0.60	0.18	0.76	0.58	0.76
trend p-value[b]		0.74	0.48	0.81	0.03	0.27
Education						
≤ middle school	52	1.15 (0.757, 1.73)	31.6 (20.3, 49.2)	0.624 (0.510, 0.763)	11.7 (8.04, 17.1)	1.03 (0.542, 1.95)
> middle school	45	1.27 (0.763, 2.12)	38.6 (22.3, 66.6)	0.686 (0.561, 0.839)	17.4 (11.6, 26.0)	1.14 (0.549, 2.35)
p-value[a]		0.75	0.57	0.50	0.16	0.84
trend p-value[b]		0.44	0.23	0.64	0.19	0.73
Income[c]						
Low	62	1.09 (0.734, 1.62)	32.8 (21.8, 49.4)	0.629 (0.526, 0.752)	12.2 (8.59, 17.4)	0.886 (0.486, 1.62)
High	35	1.43 (0.818, 2.49)	38.3 (20.3, 72.1)	0.694 (0.548, 0.879)	18.1 (11.6, 28.3)	1.52 (0.692, 3.36)
p-value[a]		0.43	0.68	0.51	0.16	0.27
trend p-value[b]		0.04	0.05	0.11	0.24	0.23
Baseline BMI						
<25 kg/m^2	59	1.33 (0.879, 2.02)	38.4 (25.0, 58.9)	0.731 (0.613, 0.872)	14.3 (9.83, 20.7)	0.969 (0.522, 1.80)
≥25 kg/m^2	38	1.03 (0.613, 1.72)	29.6 (16.5, 53.1)	0.545 (0.433, 0.687)	13.8 (9.11, 21.0)	1.27 (0.592, 2.73)
p-value[a]		0.43	0.47	0.05	0.91	0.58
trend p-value[b]		0.19	0.29	0.14	0.53	0.92
Smoking[d]						
Former/never	19	1.07 (0.407, 2.81)	32.8 (12.4, 87.1)	0.622 (0.451, 0.858)	12.1 (6.25, 23.5)	0.834 (0.319, 2.18)
Current	29	1.15 (0.67, 1.97)	40.9 (24.7, 67.6)	0.727 (0.548, 0.964)	7.83 (4.98, 12.3)	0.300 (0.157, 0.575)
p-value[a]		0.90	0.68	0.45	0.26	0.08

Characteristic	N	2,4-dichlorophenol	2,5-dichlorophenol	BPA	Methyl paraben	Propyl paraben
≥13 cigarettes/day among smokers[d]						
No	12	0.704 (0.272, 1.82)	29.3 (14.0, 61.2)	0.803 (0.467, 1.38)	7.35 (3.57, 15.1)	0.35 (0.121, 1.01)
Yes	17	1.62 (0.835, 3.15)	51.7 (24.9, 107)	0.678 (0.48, 0.957)	8.20 (4.29, 15.7)	0.269 (0.108, 0.671)
p-value[a]		0.13	0.25	0.57	0.81	0.69
Bottled water consumption[e]						
No	32	0.934 (0.581, 1.50)	21.8 (12.4, 38.6)	0.563 (0.453, 0.699)	25.4 (15.5, 41.6)	3.23 (1.32, 7.92)
Yes	17	2.39 (0.984, 5.79)	66.6 (24.6, 180)	0.752 (0.496, 1.14)	14.9 (7.90, 28.2)	1.61 (0.465, 5.58)
p-value[a]		0.06	0.05	0.21	0.18	0.35
Postmenopausal[e]						
No	25	1.41 (0.768, 2.61)	35.3 (17.2, 72.5)	0.664 (0.501, 0.880)	22.9 (12.8, 41.2)	3.80 (1.50, 9.62)
Yes	23	1.13 (0.564, 2.27)	27.7 (12.4, 61.9)	0.598 (0.442, 0.810)	18.5 (10.7, 32.1)	1.46 (0.469, 4.53)
p-value[a]		0.62	0.64	0.60	0.58	0.18
Taken medicine in past 24 hours						
No	39	1.16 (0.704, 1.91)	33.9 (20.2, 57.0)	0.682 (0.543, 0.856)	12.0 (8.09, 17.7)	0.526 (0.278, 0.996)
Yes	58	1.23 (0.805, 1.89)	35.2 (22.1, 56)	0.632 (0.527, 0.759)	15.7 (10.7, 23.0)	1.75 (0.911, 3.34)
p-value[a]		0.85	0.92	0.61	0.32	0.009
Time of day of urine collection						
Morning	31	1.37 (0.767, 2.43)	43.6 (25.5, 74.4)	0.575 (0.473, 0.699)	14.6 (8.82, 24.3)	0.837 (0.344, 2.03)
Afternoon	66	1.13 (0.766, 1.68)	31.2 (20.1, 48.4)	0.691 (0.574, 0.833)	13.8 (9.90, 19.3)	1.21 (0.686, 2.15)
p-value[a]		0.59	0.33	0.17	0.85	0.48

Samples with creatinine < 20 or > 275 mg/dL were excluded.

[a]p-value for Satterthwaite t-test. [b]p-value for continuous measure in linear regression models testing dose-response (age, education, income, and BMI only). [c]High income was defined as ≥20,000 yuan/year for females and ≥12,000 yuan/year for males. [d]Males only. [e]Females only.

Table S4. Geometric means (95% CI) of third sample urinary phenol concentrations (μg/L) by selected characteristics.

**Characteristic**	**N**	**2,4-dichlorophenol**	**2,5-dichlorophenol**	**BPA**	**Methyl paraben**	**Propyl paraben**
Sex						
Female	50	1.31 (0.916, 1.88)	30.7 (19.1, 49.5)	0.888 (0.704, 1.12)	28.8 (18.2, 45.6)	3.06 (1.48, 6.31)
Male	50	1.50 (1.00, 2.24)	47.5 (30.1, 74.9)	0.794 (0.668, 0.944)	12.8 (8.55, 19.1)	0.843 (0.467, 1.52)
p-value[a]		0.62	0.19	0.44	0.009	0.007
Age (years)						
≤50	44	1.73 (1.13, 2.64)	47.2 (27.6, 80.8)	0.945 (0.772, 1.16)	25.2 (14.9, 42.7)	3.03 (1.41, 6.49)
>50	56	1.19 (0.844, 1.68)	32.3 (21.4, 48.9)	0.765 (0.626, 0.936)	15.4 (10.6, 22.5)	0.976 (0.539, 1.77)
p-value[a]		0.17	0.26	0.14	0.13	0.02
trend p-value[b]		0.28	0.45	0.11	0.20	0.20
Education						
≤ middle school	54	1.34 (0.925, 1.93)	37.2 (23.9, 57.9)	0.831 (0.678, 1.019)	18.9 (12.5, 28.6)	1.68 (0.870, 3.26)
> middle school	46	1.48 (0.996, 2.20)	39.4 (23.8, 65.2)	0.850 (0.692, 1.05)	19.5 (11.9, 31.7)	1.52 (0.741, 3.11)
p-value[a]		0.71	0.86	0.87	0.93	0.83
trend p-value[b]		0.58	0.47	0.82	0.72	0.79
Income[c]						
Low	65	1.29 (0.906, 1.83)	33.7 (21.9, 51.9)	0.819 (0.690, 0.971)	18.6 (12.4, 27.8)	1.33 (0.725, 2.45)
High	35	1.64 (1.09, 2.47)	48.2 (29.4, 79.3)	0.881 (0.673, 1.15)	20.4 (12.3, 33.6)	2.27 (1.03, 5.01)
p-value[a]		0.36	0.28	0.65	0.77	0.28
trend p-value[b]		0.14	0.11	0.29	0.40	0.17
BMI (kg/ m^2)						
<25	56	1.34 (0.944, 1.89)	35.1 (22.5, 54.7)	0.828 (0.693, 0.988)	20.8 (13.7, 31.6)	1.90 (0.996, 3.62)
≥25	41	1.51 (0.956, 2.39)	44.4 (26.0, 75.8)	0.882 (0.687, 1.13)	17.8 (10.7, 29.7)	1.35 (0.625, 2.90)
p-value[a]		0.67	0.50	0.68	0.63	0.49
trend p-value[b]		0.88	0.81	0.71	0.68	0.89
Smoking[d]						
Former/never	19	1.48 (0.661, 3.32)	46.6 (20.3, 107.)	0.667 (0.496, 0.898)	21.5 (11.2, 41.2)	1.40 (0.455, 4.28)
Current	31	1.51 (0.946, 2.40)	48.1 (27.2, 85.0)	0.883 (0.712, 1.10)	9.28 (5.61, 15.4)	0.619 (0.309, 1.24)
p-value[a]		0.97	0.95	0.12	0.04	0.21

Characteristic	N	2,4-dichlorophenol	2,5-dichlorophenol	BPA	Methyl paraben	Propyl paraben
≥13 cigarettes/day among smokers[d]						
No	12	1.52 (0.640, 3.63)	55.2 (20.6, 148.)	0.919 (0.640, 1.32)	7.93 (3.53, 17.8)	0.568 (0.191, 1.69)
Yes	19	1.50 (0.822, 2.73)	44.1 (20.5, 94.7)	0.862 (0.642, 1.16)	10.3 (5.08, 20.7)	0.654 (0.244, 1.75)
p-value[a]		0.97	0.70	0.77	0.61	0.84
Bottled water consumption[e]						
No	33	1.38 (0.844, 2.26)	31.5 (16.2, 61.3)	0.929 (0.681, 1.27)	24.6 (13.3, 45.6)	2.89 (1.14, 7.28)
Yes	17	1.19 (0.708, 1.99)	29.3 (15.3, 55.9)	0.815 (0.563, 1.18)	38.9 (19.3, 78.7)	3.42 (0.944, 12.4)
p-value[a]		0.66	0.87	0.58	0.31	0.82
Postmenopausal[e]						
No	26	1.81 (1.12, 2.92)	38.7 (18.8, 79.9)	0.993 (0.777, 1.27)	41.1 (22.9, 73.8)	6.01 (2.25, 16.1)
Yes	23	0.870 (0.503, 1.50)	21.7 (11.3, 41.6)	0.735 (0.484, 1.12)	19.9 (9.27, 42.9)	1.49 (0.489, 4.53)
p-value[a]		0.04	0.23	0.21	0.13	0.06

Samples with creatinine < 20 or > 275 mg/dL were excluded.

[a]p-value for Satterthwaite t-test. [b]p-value for continuous measure in linear regression models testing dose-response (age, education, income, and BMI only). [c]High income was defined as ≥20,000 yuan/year for females and ≥12,000 yuan/year for males. [d]Males only. [e]Females only.

Table S5. Measures of reproducibility for urinary phenol concentrations, restricted to samples collected in the morning.

Phenol	Females: ICC (95% CI)[a]	Females: Correlation[b]	Males: ICC (95% CI)[a]	Males: Correlation[b]
Uncorrected (µg/L)				
2,4-dichlorophenol	0.42 (0.18, 0.71)	0.22	0.61 (0.40, 0.78)	0.60
2,5-dichlorophenol	0.27 (0.07, 0.65)	0.11	0.68 (0.46, 0.83)	0.50
BPA	0.09 (0.00, 0.88)	0.11	0.05 (0.00, 0.94)	0.01
Methyl paraben	0.20 (0.03, 0.65)	0.57	0.30 (0.11, 0.60)	0.75
Propyl paraben	0.09 (0.00, 0.84)	0.38	0.52 (0.30, 0.72)	0.58
Creatinine-corrected (µg/g)				
2,4-dichlorophenol	0.57 (0.33, 0.79)	0.41	0.73 (0.55, 0.85)	0.52
2,5-dichlorophenol	0.39 (0.15, 0.69)	0.15	0.79 (0.62, 0.89)	0.64
BPA	0.37 (0.13, 0.69)	0.43	0.08 (0.00, 0.76)	-0.05
Methyl paraben	0.24 (0.05, 0.65)	0.58	0.34 (0.14, 0.62)	0.71
Propyl paraben	0.14 (0.01, 0.72)	0.22	0.53 (0.32, 0.73)	0.59

Samples with creatinine < 20 or > 275 mg/dL were excluded.

Number of samples: $N_{females}$=13; N_{males}=18.

[a]Baseline sample, sample 2, and sample 3. [b]Baseline sample and the average of samples 2 and 3; values are Spearman correlation coefficients.
